# Prognostic significance of visceral pleural changes in stage IA lung adenocarcinoma: a retrospective study

**DOI:** 10.3389/fonc.2025.1658916

**Published:** 2025-09-25

**Authors:** Yingding Ruan, Yuhe You, Jianwei Han, Hongsheng Xue, Wenjun Cao, Chuan Long, Peng Sun, Yaoyu Hu, Zhilong Zhao

**Affiliations:** ^1^ Department of Thoracic Surgery, The First People’s Hospital of Jiande, Jiande, China; ^2^ Department of Thoracic Surgery, Affiliated Zhongshan Hospital of Dalian University, Dalian, China; ^3^ Department of Radiology, The First People’s Hospital of Jiande, Jiande, China; ^4^ Department of Radiology, Affiliated Zhongshan Hospital of Dalian University, Dalian, China

**Keywords:** disease-free survival, lung adenocarcinoma, overall survival, propensity score matching, visceral pleural changes

## Abstract

**Background:**

Visceral pleural changes (VPC) is increasingly detected in early-stage lung adenocarcinoma, but its clinical and prognostic significance is unclear. This retrospective multicenter study aims to evaluate the influence of VPC on OS and DFS in patients with stage IA lung adenocarcinoma.

**Methods:**

Overall, 494 patients with stage IA lung adenocarcinoma from two centers were enrolled, including 202 VPC-positive (VPC+) and 292 VPC-negative (VPC-) patients. After 1:1 propensity score matching (PSM), 284 patients (142 per group) were analyzed. The Kaplan–Meier method was used to compare survival between groups, and Cox regression analysis identified independent prognostic factors for OS and DFS.

**Results:**

Kaplan–Meier analysis showed no significant OS difference between VPC+ and VPC- group (HR 0.67, 95% CI 0.31–1.47, P = 0.320). However, DFS was significantly better in VPC+ patients compared to VPC- patients (HR 0.51, 95% CI 0.27–0.94, P = 0.028). Specifically, 5-year OS was 96.5% in VPC+ vs. 95.8% in VPC- (P = 0.845), and 5-year DFS was 95.8% in VPC+ vs. 92.3% in VPC-(P = 0.259), with no significant differences. Median OS was 76.0 months before PSM and 76.0 months after PSM. For DFS, median time was 76.0 months before PSM and 76.0 months after PSM. Cox regression identified operative time as an independent OS prognostic factor (HR 1.01, 95% CI 1.00–1.11, P = 0.039), while VPC- (HR 0.40, 95% CI 0.19–0.83, P = 0.015) and pathological stage IA3 (HR 3.12, 95% CI 1.08–9.00, P = 0.035) were independent DFS prognostic factors.

**Conclusion:**

In patients with stage IA lung adenocarcinoma, VPC- is associated with worse DFS compared to VPC+, while no significant difference in OS was observed. Pathological stage were significant prognostic factors for DFS.

## Introduction

Lung adenocarcinoma, the most common histological subtype of non-small cell lung cancer (NSCLC), constitutes a significant proportion of early-stage lung cancers (1). Patients with stage IA lung adenocarcinoma generally have a favorable prognosis, with a 5-year overall survival (OS) rate exceeding 80% (2, 3). However, the prognostic factors for stage IA lung adenocarcinoma specifically remain to be fully elucidated.

Visceral pleural invasion (VPI) is a known adverse prognostic factor in NSCLC, particularly in tumors ≤3 cm in diameter, where it predicts lymph node metastasis and postoperative recurrence (4–6). Consequently, the 8th edition of the TNM classification recommends upstaging tumors with VPI from IA to IB (7). On chest computed tomography (CT), signs such as pleural retraction, pleural traction lines, and pleural indentation are often associated with VPI (8–12). However, visceral pleural changes (VPC) do not always progress to VPI.

Recent advancements in imaging techniques have enabled the detection of subtle pleural changes. However, the clinical significance of VPC in patients with early-stage lung adenocarcinoma is still not fully understood. VPC may represent an early interaction between the tumor and the pleura, potentially influencing tumor biology and progression (9–11). The specific impact of VPC on stage IA lung adenocarcinoma remains to be further investigated.

This retrospective analysis, including 494 patients, aims to explore the prognostic significance of VPC in stage IA lung adenocarcinoma and determine if it can serve as an independent prognostic factor for OS and disease-free survival (DFS).

## Patients and methods

### Study population and eligibility criteria

This retrospective study was based on the data of patients who underwent surgical resection for lung cancer at The First People’s Hospital of Jiande (Jiande, China) and Affiliated Zhongshan Hospital of Dalian University (Dalian, China) from January 2012 to December 2018. All patients underwent preoperative high-resolution chest CT (1–1.25 mm). CT images were acquired in the supine position during inspiratory breath-hold. The inclusion criteria were (1) patients who underwent lung resection and were diagnosed with lung adenocarcinoma and (2) patients with a tumor size of ≤3 cm. The exclusion criteria were (1) prior lung resection surgery; (2) non-primary pulmonary malignancies; (3) transfer to other hospitals; (4) non-adenocarcinoma histopathology; (5) pathologically confirmed adenocarcinoma *in situ* or minimally invasive adenocarcinoma; (6) non-stage IA lung adenocarcinoma; (7) loss to follow-up; (8) palliative surgery; (9) centrally located lung cancer; (10) Neoadjuvant therapy with preoperative radiotherapy or chemotherapy; and (11) conversion to thoracotomy during surgery.

All pathological diagnoses were based on hospital pathology reports. All patients were restaged according to the 8th edition of the TNM classification established by the International Association for the Study of Lung Cancer (7).

The study adhered to the principles outlined in the Declaration of Helsinki and was approved by the Ethics Committee of The First People’s Hospital of Jiande (Ethics Committee Approval Number: 20250523-KY-002-01). The requirement for written informed consent was waived due to the retrospective nature of the study. To protect patient privacy, all personal identifiers were removed from the dataset before analysis, and only de-identified data were used. The original data were accessible only to the authors of the study. Furthermore, data access was restricted to the research team, and all data were stored securely.

### Data collection

The following data were retrospectively collected: demographic characteristics (sex, age, body mass index, and smoking history), comorbidities (hypertension, diabetes mellitus, coronary heart disease, and chronic obstructive pulmonary disease), VPC, density classification, nodule depth, TNM stage, surgical approaches, resection site, type of lung resection, total number of lymph nodes retrieved, number of mediastinal lymph nodes retrieved, total lymph node stations explored, mediastinal lymph node stations explored, surgical duration, intraoperative blood loss volume, drainage time and volume, length of postoperative hospital stay, OS, and DFS.

### Pulmonary nodule imaging characteristics and VPC

Pure ground-glass nodules (pGGN) were characterized by a mild, uniform increase in lung tissue density on chest CT, presenting with a translucent, frosted-glass appearance. The internal vascular and bronchial structures remained clearly visible, and there were no solid components. Mixed ground-glass nodules (mGGN), or part-solid nodules, were those that contained both ground-glass density and solid components, with the solid portion having a higher density (0 < consolidation-to-tumor ratio [CTR] < 1), which may obscure vascular and bronchial structures. Solid nodules (SNs) were those composed entirely of solid components of uniform density, lacking ground-glass or cystic elements, and with a CT value close to that of soft tissue (CTR = 1) (13).

**Figure 1:**
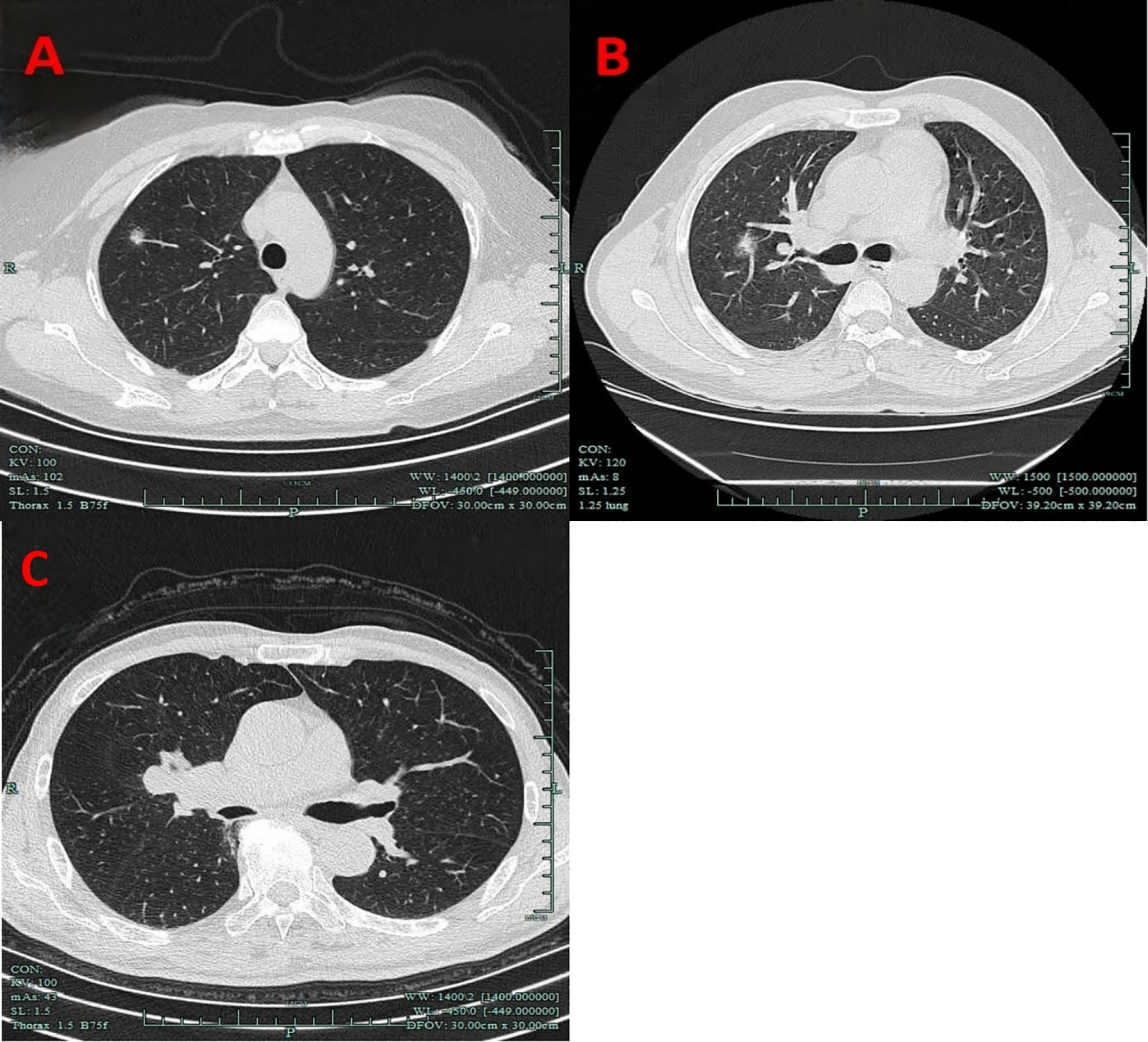
Imaging manifestations of nodule depth. **(A)** The outer one-third. **(B)** The inner two-thirds. **(C)** Centrally located lung masses.

Nodule depth was defined as the distance from the pulmonary nodule to the pleura, relative to the distance from the pleura to the hilum, categorized into the outer one-third and the inner two-thirds, and it did not include centrally located lung masses (**Figure 1**).

**Figure 2:**
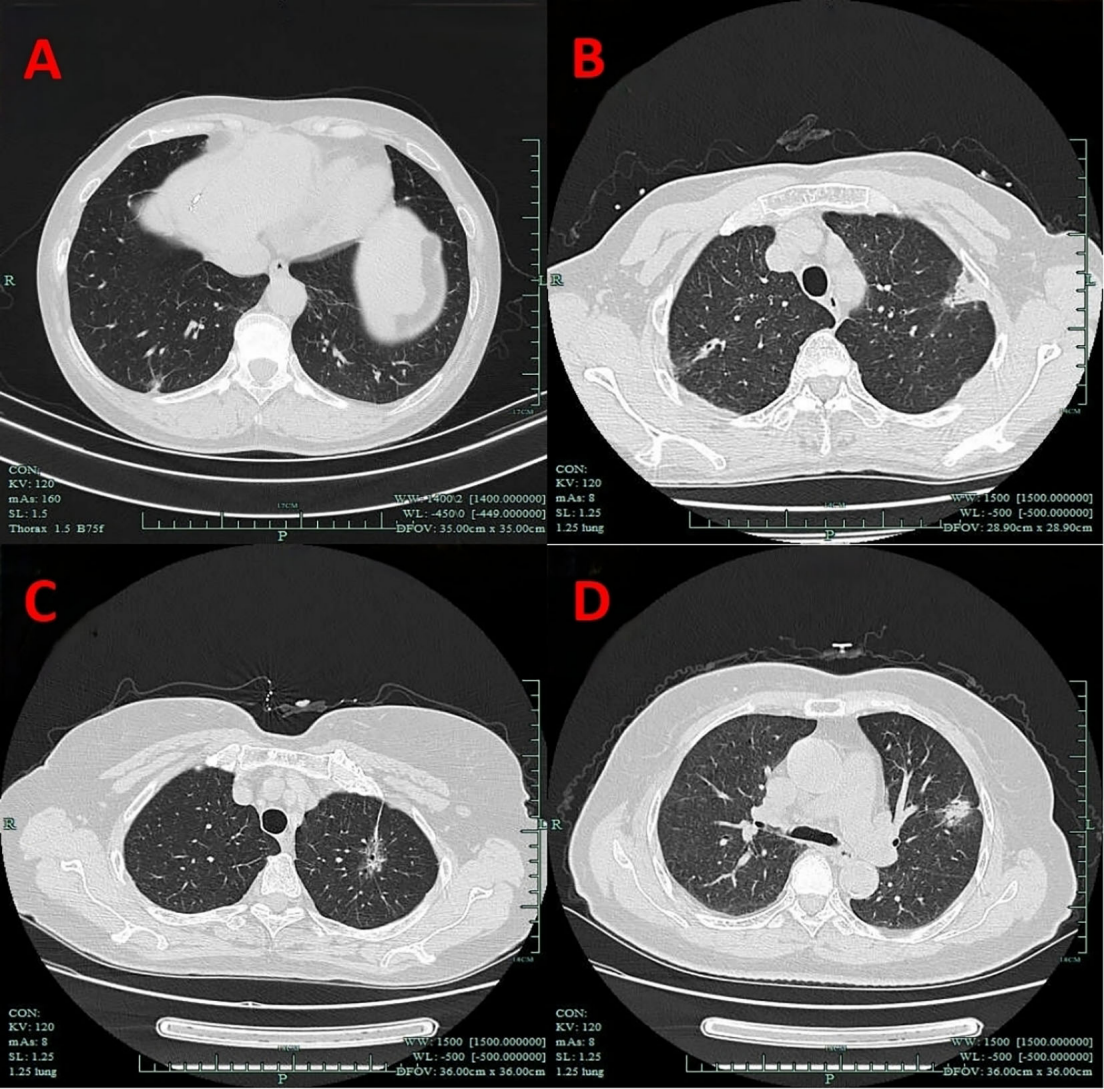
Imaging manifestations of VPC. **(A)** Pleural indentation. **(B)** Pleural attachment. **(C)** Pleural traction. **(D)** Pleural tail sign. VPC, visceral pleural changes.

VPC represent a broad concept that includes any form of pleural involvement, such as thickening or adhesion, without penetrating the elastic layer or invading the visceral pleura (14). The key imaging manifestations of VPC include pleural traction, pleural tail sign, pleural attachment, and pleural indentation (**Figure 2**). In this study, VPC referred exclusively to visceral pleural involvement without visceral pleural invasion (VPI).

Radiological assessments for both nodule characteristics and VPC were independently conducted by a thoracic surgeon and a thoracic radiologist from each institution. For patients with uncertain findings, a consensus was reached through joint discussion.

### Follow-up

Follow-up data were collected through retrospective analysis of patients’ imaging records from their respective hospitals. A designated thoracic surgeon and a thoracic radiologist systematically reviewed postoperative imaging to detect tumor recurrence, encompassing both locoregional recurrence and distant metastasis, and documented the timing of each follow-up. For patients who were lost to follow-up or chose to undergo postoperative imaging at other hospitals, a standardized telephone follow-up system was used to collect the survival data.

OS was defined as the time from the date of diagnosis until death from any cause or until March 2025, whichever came first. DFS was defined as the time from the date of diagnosis to the first event of disease progression, whether locoregional or distant; death from any cause; or until March 2025, whichever came first.

### Statistical analysis

We conducted PSM at a 1:1 ratio to enhance the intergroup comparability and reduce potential bias. Propensity scores were calculated using a logistic regression model that included age, density classification, nodule depth, TNM stage, total number of lymph nodes retrieved, and total lymph node stations explored. We used the nearest-neighbor matching method with a caliper value of 0.2 and without replacement to perform the PSM. This approach helped to balance the distribution of potential confounders between the groups (P > 0.05).

To evaluate the balance in the covariates between the groups after PSM, we used the standardized mean difference (SMD). Generally, an SMD of <0.10 indicates good balance, 0.10–0.34 suggests minor imbalance, 0.35–0.64 indicates moderate imbalance, 0.65–1.19 indicates substantial imbalance, and ≥1.20 reflects very large imbalance. Our results showed that all covariates were within the acceptable range for balance.

For normally distributed continuous data, the Student’s t-test was used for comparisons between groups, and the data are presented as the mean ± standard deviation. For non-normally distributed continuous data, the Wilcoxon rank-sum test was used for comparisons, and the data are presented as the median with interquartile range (25th–75th percentiles). Categorical variables were compared using the chi-square test or Fisher’s exact test, as appropriate, and are presented as percentages.

Kaplan–Meier plots were used for the univariable analysis of OS and DFS, and the log-rank test was used for survival comparisons between the groups. Variables significant at P < 0.05 in the univariable analysis were included in the multivariable Cox proportional-hazards regression analysis to determine independent prognostic factors.

All analyses were performed using GraphPad Prism 8 and R (R Foundation for Statistical Computing, Vienna, Austria).

## Results

### Patient characteristics and perioperative results

**Figure 3:**
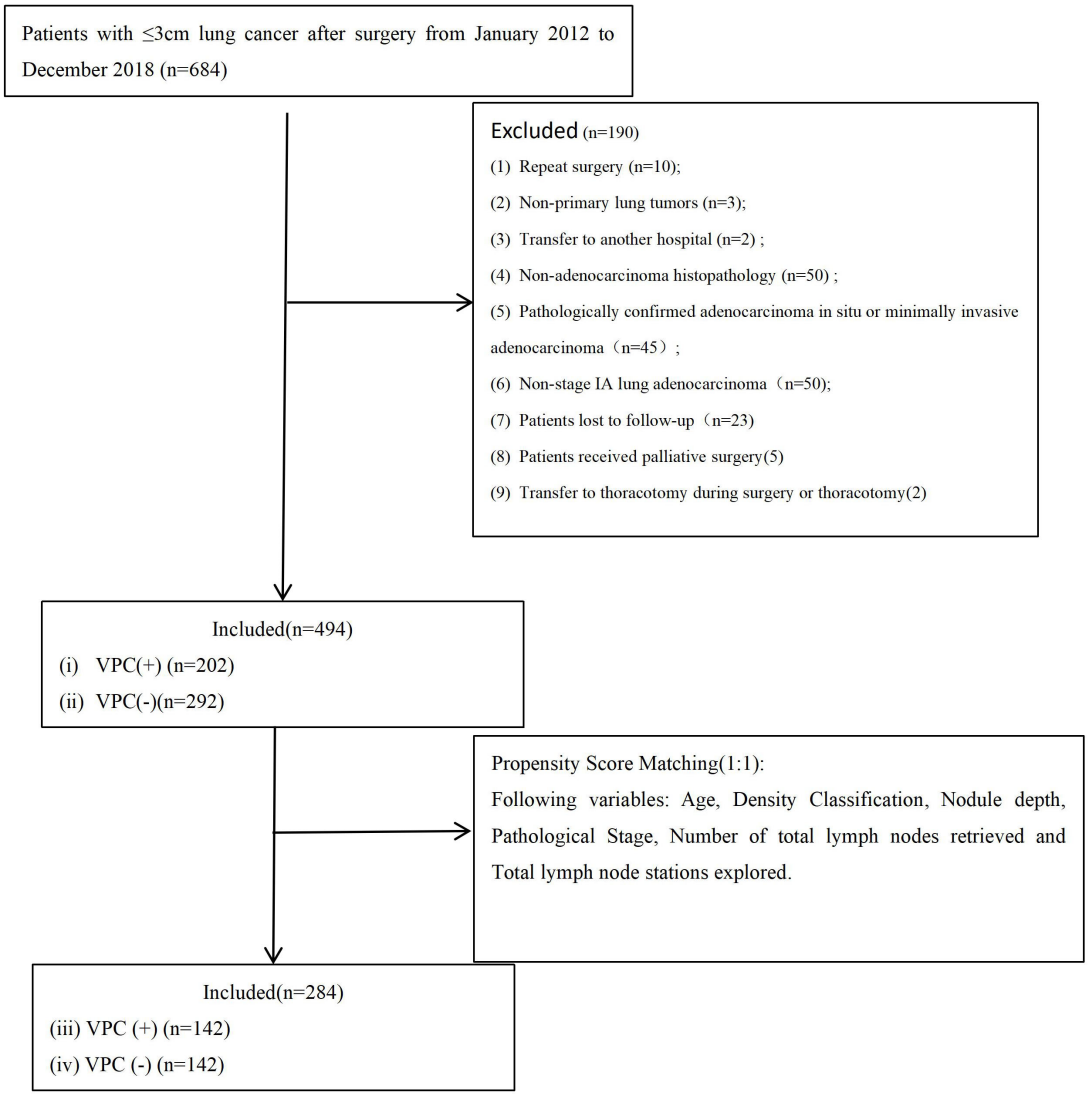
Flowchart of patient selection. VPC+, visceral pleural changes-positive; VPC−, visceral pleural changes-negative.

From January 2012 to December 2018, 684 patients underwent VATS for lung cancer with a tumor size of ≤3 cm at The First People’s Hospital of Jiande (n = 309) or the Affiliated Zhongshan Hospital of Dalian University (n = 375). After excluding 190 patients, 494 were included in the final analysis (**Figure 3**). The cohort included 202 patients with VPC and 292 without VPC. The nodule types were classified as pGGN in 191 patients (38.7%), mGGN in 187 (37.9%), and SN in 116 (23.4%). The surgical approaches were uniportal VATS (U-VATS) in 286 patients (57.9%) and multiportal VATS (M-VATS) in 208 patients (42.1%). The tumor location was peripheral (outer one-third lung zone) in 294 patients (59.5%) and central (inner two-thirds perihilar region) in 200 patients (40.5%). The median OS time was 76.0 months (interquartile range (IQR): 73.0 - 83.75 months) and 76.0 months (IQR: 73.0 - 83.0 months) for DFS.

After 1:1 PSM, the data of 284 patients (142 with and 142 without VPC) were analyzed (**Figure 3**). The matched subgroups comprised 71 patients with pGGN (25.0%), 136 with mGGN (47.9%), and 77 with SN (27.1%). The surgical approaches were U-VATS in 159 patients (56.0%) and M-VATS in 125 patients (44.0%). The tumor location remained peripheral in 171 patients (60.2%) and central in 113 patients (39.8%). The post-PSM The median OS time was 76.0 months (IQR: 73.0 - 84.25 months) and 76.0 months (IQR: 73.0 - 84.0 months) for DFS. All preoperative variables were balanced between the groups (P > 0.05). The baseline characteristics of the patients are summarized in **Table 1**.

**Table 1:** Patients’ characteristics before and after PSM in the VPC+ and VPC− groups.

Variables	Total(n=494)	Before PSM			Total(n=284)	After PSM			SMD
		VPC(+)(n=202)	VPC(-)(n=292)	P-Value		VPC(+)(n=142)	VPC(-)(n=142)	P-Value	
Sex, n(%)				0.545				0.109	0.191
Male	166 (33.6)	71 (35.2)	95 (32.5)		103 (36.3)	45 (31.7)	58 (40.9)		
Female	328 (66.4)	131 (64.8)	197 (67.5)		181 (63.7)	97 (68.3)	84 (59.1)		
Smoking, n(%)	103 (20.9)	49 (24.3)	54 (18.5)	0.212	70 (24.7)	33 (23.2)	37 (26.1)	0.582	0.065
Age(Years)	60.55 ± 10.48	62.42 ± 10.79	59.25 ± 10.08	0.001	61.06 ± 10.76	61.29 ± 11.43	60.83 ± 10.08	0.721	0.043
BMI(kg/m^2^)	23.69 ± 3.53	23.32 ± 3.59	23.94 ± 3.47	0.058	23.67 ± 3.63	23.35 ± 3.70	23.99 ± 3.54	0.142	0.175
Comorbidities, n(%)	193 (39.1)	87 (43.1)	106 (36.3)	0.130	122 (43.0)	62 (43.7)	60 (42.3)	0.811	0.028
Density Classification, n(%)				<0.001				0.998	0.028
pGGN	191 (38.7)	35 (17.3)	156 (53.4)		71 (25.0)	35 (24.7)	36 (25.4)		
mGGN	187 (37.9)	100 (49.5)	87 (29.8)		136 (47.9)	69 (48.6)	67 (47.2)		
SN	116 (23.4)	67 (33.2)	49 (16.8)		77 (27.1)	38 (26.7)	39 (27.4)		
Nodule depth, n(%)				<0.001				0.904	0.014
Pulmonary mass located in outer 1/3 lung zone	294 (59.5)	146 (72.3)	148 (50.7)		171 (60.2)	86 (60.6)	85 (59.9)		
Pulmonary mass located in inner 2/3 perihilar region	200 (40.5)	56 (27.7)	144 (49.3)		113 (39.8)	56 (39.4)	57 (40.1)		
TNM Stage*, n(%)				<0.001				0.775	0.047
IA1	173 (35.0)	51 (25.2)	122 (41.8)		99 (34.9)	51 (35.9)	48 (33.8)		
IA2	248 (50.2)	110 (54.5)	138 (47.2)		141 (49.7)	69 (48.6)	72 (50.7)		
IA3	73 (14.8)	41 (20.3)	32 (11.0)		44 (15.4)	22 (15.5)	22 (15.5)		
Resection Site, n(%)				0.535				0.782	0.200
Right upper	180 (36.4)	67 (33.2)	113 (38.7)		106 (37.3)	50 (35.2)	56 (39.4)		
Right middle	37 (7.5)	18 (8.9)	19 (6.5)		17 (6.0)	8 (5.6)	9 (6.3)		
Right lower	96 (19.4)	44 (21.8)	52 (17.8)		55 (19.4)	33 (23.3)	22 (15.5)		
Lef upper	117 (23.7)	46 (22.8)	71 (24.3)		72 (25.3)	34 (23.9)	38 (26.8)		
Lef lower	64 (13.0)	27 (13.3)	37 (12.7)		34 (12.0)	17 (12.0)	17 (12.0)		
Approaches, n(%)								0.550	0.071
U-VATS	286 (57.9)	101 (50.0)	185 (63.4)	0.003	159 (56.0)	77 (54.2)	82 (57.8)		
M-VATS	208 (42.1)	101 (50.0)	107 (36.6)		125 (44.0)	65 (45.8)	60 (42.2)		
Type of lung resection, n(%)				0.064				0.727	0.075
Lobectomy	369 (74.7)	160 (79.2)	209 (71.6)		214 (75.4)	106 (74.7)	108 (76.1)		
Segmental	77 (15.6)	25 (12.4)	52 (17.8)		45 (15.9)	22 (15.5)	23 (16.2)		
Wedge	48 (9.7)	17 (8.4)	31 (10.6)		25 (8.7)	14 (9.8)	11 (7.7)		
Operative time (min)	140.00 (100.00, 183.00)	153.00 (105.00, 200.00)	130.00 (95.00, 180.00)	0.005	145.50 (101.50, 191.00)	153.50 (105.00, 201.50)	135.00 (100.00, 180.00)	0.151	0.176
Intraoperative bleeding volume (ml)	50.00 (50.00, 100.00)	50.00 (50.00, 100.00)	50.00 (50.00, 100.00)	<0.001	50.00 (50.00, 100.00)	50.00 (50.00, 100.00)	50.00 (50.00, 100.00)	0.019	0.382
Number of total lymph nodes retrieved	8.00 (3.00, 14.00)	9.00 (4.00, 15.75)	8.00 (2.00, 14.00)	0.032	8.50 (3.00, 15.00)	7.00 (3.00, 15.00)	9.00 (3.00, 15.00)	0.517	0.036
Number of mediastinal lymph nodes retrieved	4.00 (0.00, 9.00)	5.00 (1.00, 9.00)	3.00 (0.00, 8.00)	0.004	4.00 (0.00, 9.00)	4.00 (0.00, 9.00)	5.00 (0.00, 9.00)	0.832	0.016
Total lymph node stations explored	4.00 (2.00, 5.00)	4.00 (2.00, 5.00)	3.00 (1.00, 5.00)	0.021	4.00 (2.00, 5.00)	3.00 (2.00, 5.00)	4.00 (2.00, 5.00)	0.491	0.078
Mediastinal lymph node stations explored	2.00 (0.00, 3.00)	2.00 (1.00, 3.00)	2.00 (0.00, 3.00)	<0.001	2.00 (0.00, 3.00)	2.00 (0.00, 3.00)	2.00 (0.00, 3.00)	0.951	0.022
Drainage volume(ml)	700.00 (406.25, 1147.50)	860.00 (550.00, 1342.50)	600.00 (360.00, 1002.50)	<0.001	800.00 (450.00, 1250.00)	860.00 (502.50, 1285.00)	775.00 (422.75, 1230.00)	0.399	0.016
Drainage time (min)	4.00 (3.00, 6.00)	4.00 (3.00, 6.75)	3.00 (3.00, 5.00)	0.009	4.00 (3.00, 6.00)	4.00 (3.00, 7.00)	4.00 (3.00, 6.00)	0.677	0.031
Postoperative complications, n(%)	56 (11.3)	25 (12.4)	31 (10.6)	0.544	41 (14.44)	17 (11.97)	24 (16.90)	0.237	0.141
Postoperative hospital stay (day)	6.52 (5.00, 9.39)	7.00 (5.00, 10.43)	6.00 (5.00, 8.45)	0.002	7.00 (5.00, 10.00)	7.00 (5.00, 10.91)	6.45 (5.00, 9.29)	0.309	0.102

BMI, Body mass index; M-VATS, Multiportal video-assisted thoracoscopic surgery; VPC(+), Visceral pleural changes positive; VPC(-), Visceral pleural changes negative; pGGN, Prue Ground Glass Nodule; mGGN, Mixed Ground Glass Nodule; SN, Solid Nodule; PMS, Propensity Score Matching; U-VATS, Uniportal video-assisted thoracoscopic surgery; *8th edition TNM stage grouping. Data are presented as n (%), mean ± standard deviation, or M (P25, P75).

### Prognostic factor analysis

The univariable analysis revealed associations of OS with age (P = 0.036), pathological stage (IA3) (P = 0.047) and number of total lymph nodes retrieved (P = 0.038). DFS was significantly associated with pleural indentation status (P = 0.031), smoking (P = 0.040), and pathological stage (IA3) (P = 0.001) (**Table 2**).

**Table 2:** Univariate analysis of the factors influencing OS and DFS in patients with lung adenocarcinoma.

Variables	OS				DFS			
	Estimate	S.E	P	HR (95% CI)	Estimate	S.E	P	HR(95% CI)
VPC								
-	Ref				Ref			
+	-0.40	0.40	0.321	0.77(0.31, 1.47)	-0.68	0.32	0.031	0.51 (0.27, 0.94)
Sex								
Male	Ref				Ref			
Female	-0.29	0.42	0.485	0.75 (0.33, 1.69)	-0.13	0.32	0.677	0.88 (0.47, 1.64)
Smoking	0.76	0.43	0.076	2.14 (0.92, 4.95)	0.67	0.33	0.040	1.95 (1.03, 3.68)
Age	0.05	0.02	0.036	1.05 (1.00, 1.10)	0.03	0.02	0.073	1.03 (0.99, 1.06)
BMI	0.04	0.06	0.493	1.04 (0.93, 1.16)	0.01	0.04	0.764	1.01 (0.93, 1.10)
Comorbidities	0.59	0.41	0.148	1.80 (0.81, 4.01)	0.14	0.31	0.642	1.15 (0.63, 2.09)
Density Classification								
pGGN	Ref				Ref			
mGGN	-0.37	0.49	0.443	0.69 (0.27, 1.79)	0.04	0.43	0.932	1.04 (0.45, 2.39)
SN	-0.16	0.54	0.767	0.85 (0.30, 2.45)	0.62	0.43	0.147	1.87 (0.80, 4.34)
Nodule depth								
Pulmonary mass located in outer 1/3 lung zone	Ref				Ref			
Pulmonary mass located in inner 2/3 perihilar region	-0.47	0.42	0.258	0.62 (0.27, 1.41)	0.12	0.31	0.699	1.13 (0.62, 2.05)
TNM Stage*								
IA1	Ref				Ref			
IA2	0.42	0.49	0.398	1.52 (0.58, 4.00)	0.65	0.41	0.118	1.91 (0.85, 4.29)
IA3	1.11	0.56	0.047	3.05 (1.01, 9.15)	1.45	0.45	0.001	4.24 (1.77, 10.17)
Resection Site								
Right upper	Ref				Ref			
Right middle	-0.40	1.05	0.703	0.67 (0.09, 5.26)	0.28	0.63	0.657	1.32 (0.38, 4.59)
Right lower	-0.41	0.60	0.495	0.67 (0.21, 2.14)	-0.22	0.46	0.638	0.81 (0.33, 1.98)
Lef upper	0.44	0.47	0.350	1.55 (0.62, 3.86)	0.37	0.38	0.332	1.45 (0.69, 3.06)
Lef lower	-0.96	0.80	0.230	0.38 (0.08, 1.84)	-0.08	0.50	0.873	0.92 (0.35, 2.44)
Approaches								
U-VATS	Ref				Ref			
M-VATS	-0.12	0.45	0.800	0.89 (0.37, 2.17)	0.02	0.35	0.948	1.02 (0.52, 2.01)
Type of lung resection								
Lobectomy	Ref				Ref			
Segmental	-1.11	1.03	0.280	0.33 (0.04, 2.48)	-1.75	1.02	0.086	0.17 (0.02, 1.28)
Wedge	0.71	0.55	0.200	2.03 (0.69, 6.01)	0.33	0.48	0.496	1.39(0.54, 3.55)
Operative time	0.00	0.00	0.432	1.00 (0.99, 1.01)	0.00	0.00	0.204	1.00 (0.99, 1.01)
Intraoperative bleeding volume	0.00	0.00	0.536	1.00 (0.99, 1.00)	0.00	0.00	0.628	1.00 (0.99, 1.00)
Number of total lymph nodes retrieved	-0.06	0.031	0.038	0.94 (0.88, 0.99)	-0.03	0.02	0.151	0.97 (0.93, 1.01)
Number of mediastinal lymph nodes retrieved	-0.071	0.04	0.107	0.932 (0.86, 1.02)	-0.03	0.03	0.371	0.98 (0.92, 1.03)
Total lymph node stations explored	-0.11	0.09	0.240	0.90 (0.75, 1.07)	-0.06	0.07	0.424	0.95 (0.83, 1.08)
Mediastinal lymph node stations explored	-0.12	0.13	0.369	0.89 (0.68, 1.15)	-0.02	0.10	0.877	0.99 (0.81, 1.20)
Drainage volume	0.00	0.00	0.404	1.00 (1.00, 1.00)	0.00	0.00	0.319	1.00 (1.00, 1.00)
Drainage time	0.04	0.04	0.389	1.04 (0.95, 1.13)	0.01	0.04	0.720	1.01 (0.94, 1.09)
Postoperative complications	-0.39	0.62	0.527	0.68 (0.20, 2.27)	-0.36	0.48	0.451	0.70 (0.27, 1.78)
Postoperative hospital stay	0.01	0.04	0.872	1.01 (0.93, 1.09)	-0.00	0.03	0.957	0.99 (0.94, 1.06)

BMI, Body mass index; DFS, Disease- free survival; HR, Hazard ratio; M-VATS, Multiportal video-assisted thoracoscopic surgery; OS, Overall survival; VPC(+), Visceral pleural changes positive; VPC(-), Visceral pleural changes negative; pGGN, Prue Ground Glass Nodule; mGGN, Mixed Ground Glass Nodule; SN, Solid Nodule; S.E, Standard error; U-VATS, Uniportal video-assisted thoracoscopic surgery; *8th edition TNM stage grouping.

Multivariable Cox proportional-hazards regression identified number of total lymph nodes retrieved (hazard [HR] 0.92, 95% confidence interval [CI] 0.87–0.99, P = 0.027) as an independent predictor of OS. VPC+ status (HR 0.49, 95% CI 0.27–0.91, P = 0.025) and IA3 (HR 4.06, 95% CI 1.67–9.87, P = 0.002) were independent predictors of DFS (**Table 3**).

**Table 3:** Multivariable analysis of the factors influencing OS and DFS in patients with lung adenocarcinoma.

Variables	OS				DFS			
	Estimate	S.E	P	HR (95% CI)	Estimate	S.E	P	HR(95% CI)
Smoking					0.49	0.33	0.140	1.63 (0.85, 3.10)
Age	0.03	0.02	0.116	1.03 (0.99, 1.08)	0.04	0.02	0.071	1.04 (1.00, 1.08)
Number of total lymph nodes retrieved	-0.07	0.03	0.027	0.93 (0.87, 0.99)				
VPC								
-					Ref			
+					-0.71	0.32	0.025	0.49 (0.27, 0.91)
TNM Stage*								
IA1	Ref				Ref			
IA2	0.50	0.50	0.318	1.65 (0.62, 4.40)	0.60	0.42	0.150	1.82 (0.81, 4.11)
IA3	1.13	0.59	0.056	3.09 (0.97, 9.78)	1.40	0.45	0.002	4.06 (1.67, 9.87)

DFS, Disease- free survival; HR, Hazard ratio; OS, Overall survival; VPC(+), Visceral pleural changes positive; VPC(-), Visceral pleural changes negative; S.E, Standard error; *8th edition TNM stage grouping.

### Survival analysis

**Figure 4:**
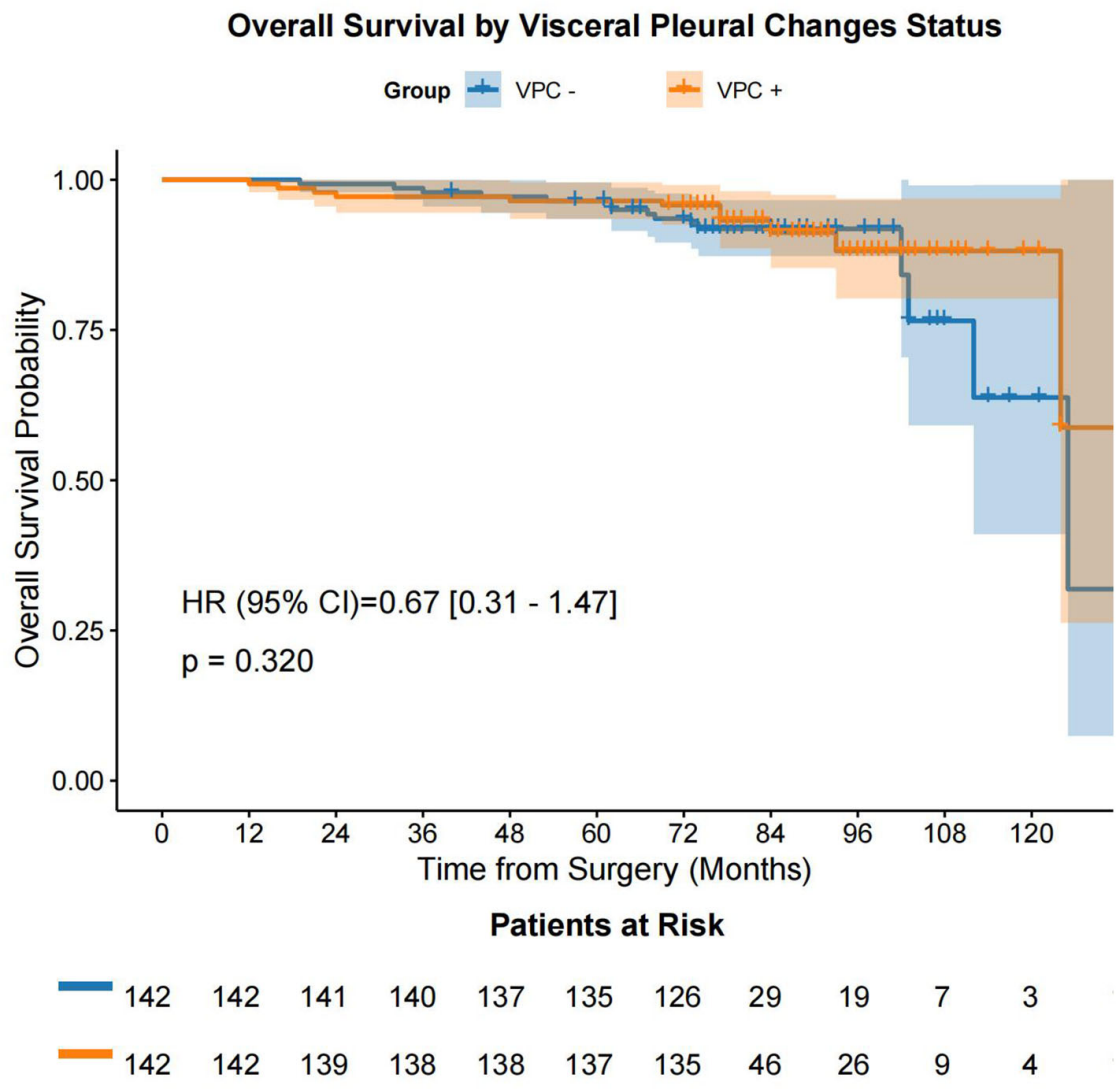
OS in the VPC+ and VPC− groups. OS, overall survival; VPC+, visceral pleural changes-positive; VPC−, visceral pleural changes-negative.

**Figure 5:**
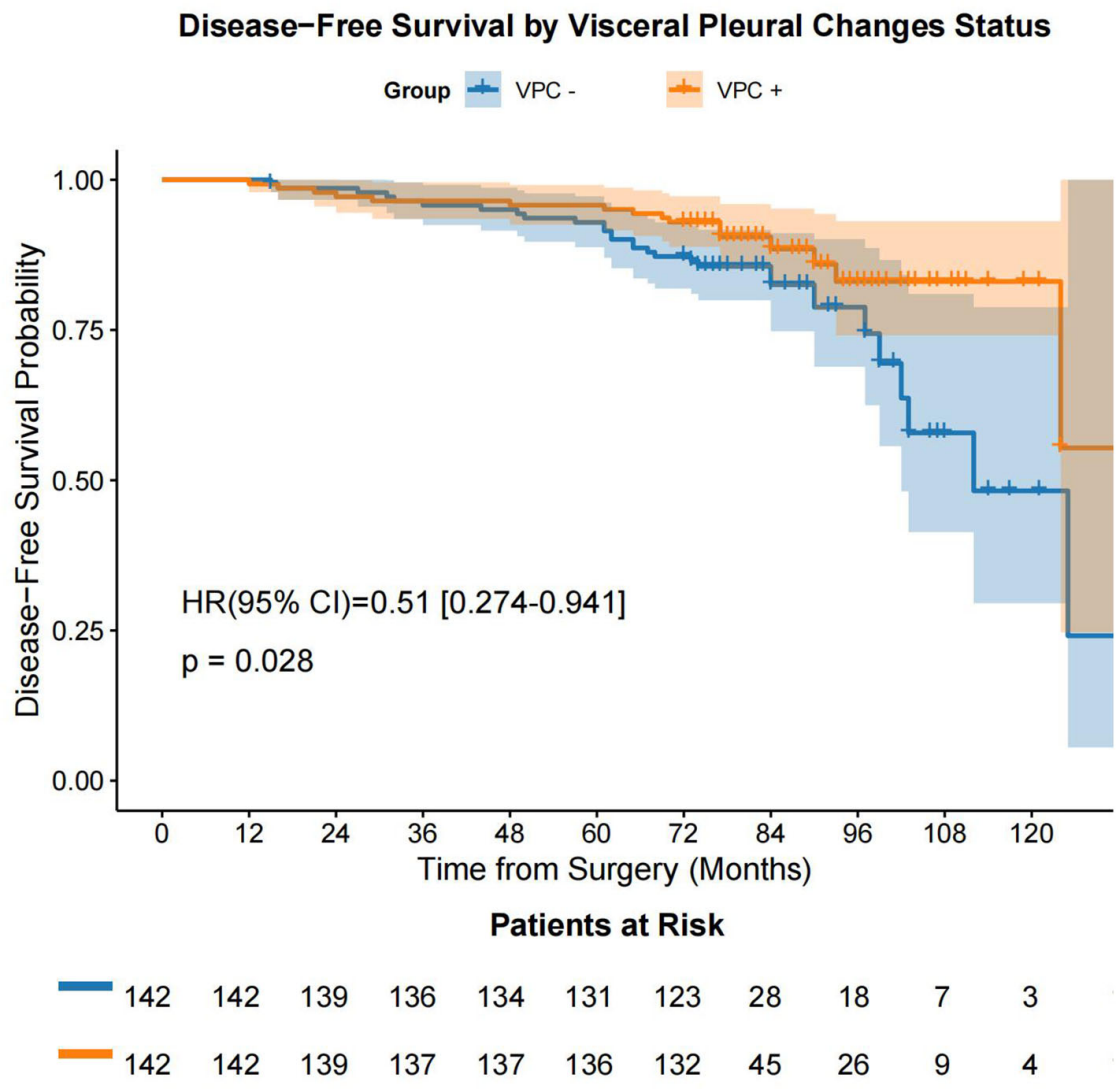
DFS in the VPC+ and VPC− groups. DFS, disease-free survival; VPC+, visceral pleural changes-positive; VPC−, visceral pleural changes-negative.

Following the prognostic factor analysis, survival analysis was performed. After PSM, VPC-positive (VPC+) and VPC-negative (VPC−) patients had median OS durations of 77.0 months (IQR: 74.0 - 90.0 months) and 75.0 months (IQR: 73.0 - 78.75 months), respectively (HR 0.67, 95% CI 0.31-1.47, P = 0.320), and median DFS durations of 77.0 months (IQR: 74.0 - 89.0 months) months and 75.0 months (IQR: 73.0 - 78.0 months), respectively (HR 0.51, 95% CI 0.27-0.94, P = 0.028) (**Figure 4**, **Figure 5**). No significant differences were found in the rates of 5-year OS (97.2% vs. 97.9%, respectively, P > 0.05) or 5-year DFS (97.2% vs. 95.1%, respectively, P = 0.541) between the VPC+ and VPC− groups.

### Subgroup survival analysis

**Figure 6:**
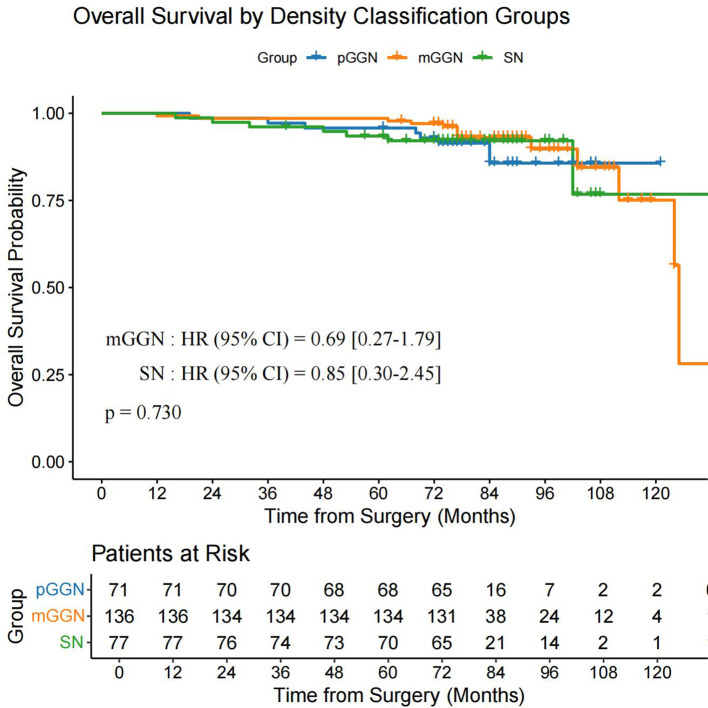
OS in the pGGN, mGGN, and SN groups. mGGN, mixed ground-glass nodules; OS, overall survival; pGGN, pure ground-glass nodules; SN, solid nodules.

**Figure 7:**
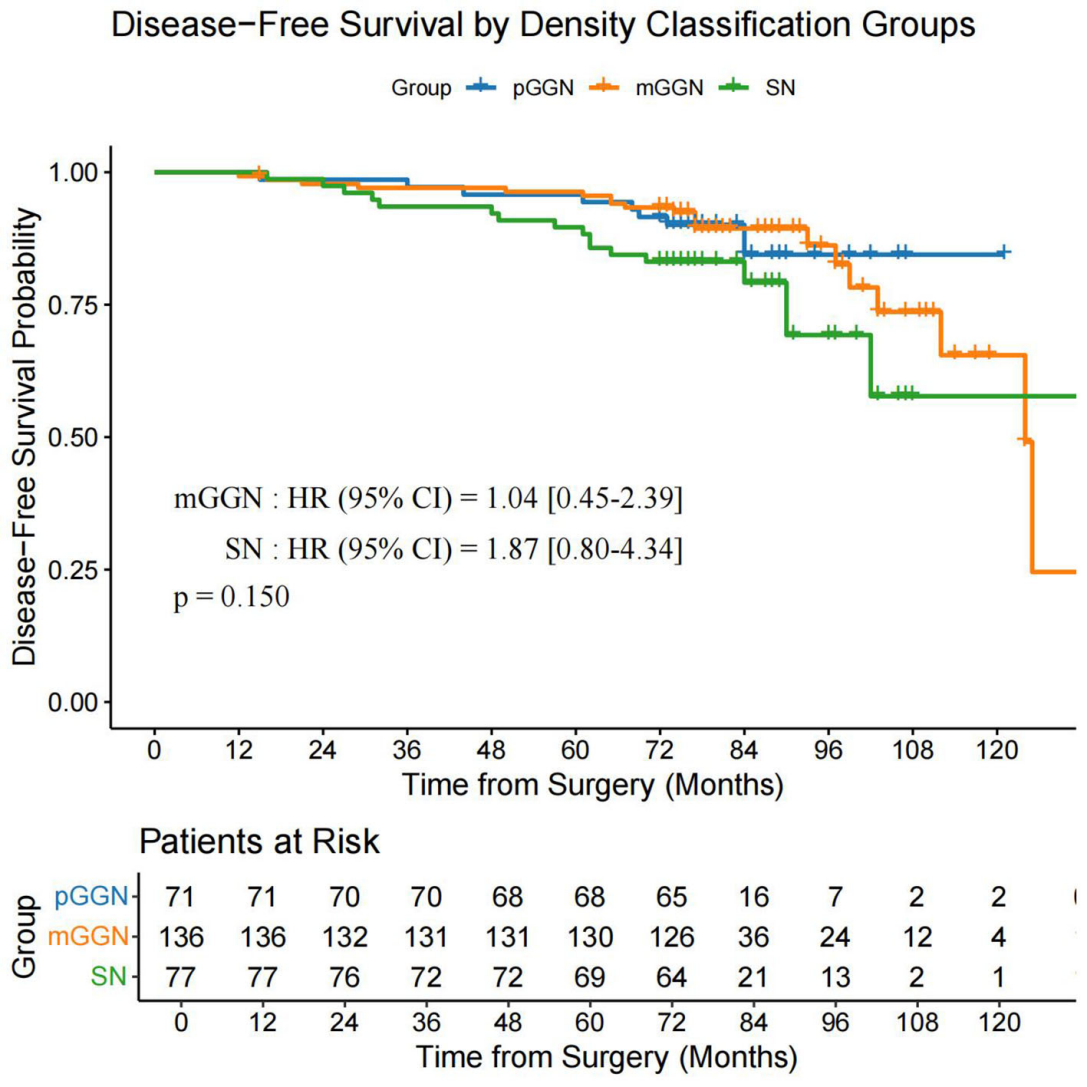
DFS in the pGGN, mGGN, and SN groups. DFS, disease-free survival; pGGN, pure ground-glass nodules; mGGN, mixed ground-glass nodules; SN, solid nodules.

Using the data after PSM, we performed a subgroup survival analysis by categorizing patients into three groups: pGGN (n = 71), mGGN (n = 136), and SN (n = 77). The analysis revealed no significant differences in OS (P = 0.730) and DFS (P = 0.150) among the three groups (**Figure 6**, **Figure 7**).

## Discussion

This study investigated the prognostic significance of VPC on DFS and OS in patients with stage IA lung adenocarcinoma. After PSM, the median follow-up duration for both groups of patients exceeded 6 years. The results suggested that VPC status may influence DFS but not OS. Specifically, patients with VPC+ status had a longer mean DFS than those without VPC (VPC−), although no significant difference in OS was observed between the two groups. Age emerged as an independent prognostic factor for OS, while VPC status and pathological stage (IA3) were identified as independent prognostic factors for DFS. Furthermore, no significant differences in 5-year OS and DFS were found between the VPC+ and VPC− groups.

In previous studies, VPI has been considered as a poor prognostic factor in patients with lung cancer (15–20). However, two studies from China indicated that in stage I NSCLC, VPI is not a prognostic factor (21, 22). These findings highlight the significant heterogeneity in the involvement of the visceral pleura in lung cancer prognosis. In contrast, few studies have evaluated the prognostic role of VPC in early-stage lung adenocarcinoma. Unlike VPI, which promotes tumor progression and lymph node metastasis, VPC may indicate an early, non-invasive interaction between the tumor and the pleura. Histopathologically, VPC may be associated with reactive fibrosis, inflammatory changes, or thickened septal edema, rather than true invasion (23–25). This distinction is crucial, as it suggests that VPC might represent a distinct biological interaction between the tumor and pleura that does not necessarily progress to invasion.

A previous study (26) showed that in T1-stage patients, those with pleural contact had significantly worse 3-year cause-specific mortality and OS rates than those without pleural contact (17.6% [95% CI 10.7%–25.9%] vs. 6.6% [95% CI 3.5%–11.1%], P < 0.01, and 58.2% [95% CI 47.6%–67.5%] vs. 77.6% [95% CI 70.5%–83.2%], P < 0.01, respectively). Multivariate analysis indicated that pleural contact was associated with cause-specific mortality (HR 1.96, 95% CI 1.09–3.52, P = 0.03) and OS (HR 1.59, 95% CI 1.08–2.34, P = 0.02), suggesting that pleural contact is linked to significantly worse survival in patients with clinical T1N0M0 lung cancer. Unlike the extensively studied VPI, our study suggested that VPC also has clinical relevance. A key distinction is that VPC do not always progress to VPI. In the present study, we included patients with VPC, but we excluded patients with VPI. The results showed that VPC and pathological stage were independent prognostic factors for DFS (VPC: HR 0.49, 95% CI 0.27–0.91, P = 0.025; IA3: HR 4.06, 95% CI 1.67–9.87, P = 0.002), but not for OS. The Kaplan–Meier analysis revealed no significant difference in OS between the VPC+ and VPC− groups (P = 0.320), but there was a significant difference in DFS between the groups (P = 0.028). This supports VPC as an early indicator of tumor-pleura interaction impacting DFS.

The observed DFS advantage (P = 0.025) without a corresponding OS benefit (P = 0.320) in VPC+ patients warrants consideration. A key consideration is the potential impact of effective salvage therapies following recurrence. In contemporary oncology practice, patients with recurrent lung adenocarcinoma, particularly those with targetable mutations or PD-L1-positive disease, have access to increasingly effective post-recurrence treatments, including targeted agents and immunotherapy, which demonstrably improve survival outcomes in advanced settings (27, 28). Patients in the VPC− group experienced recurrence earlier and thus had greater opportunity to receive and benefit from these potent salvage options. This may have substantially extended their survival, thereby mitigating the OS advantage potentially conferred by the prolonged DFS in the VPC+ group. The later recurrence timing in the VPC+ cohort could also dilute the measurable OS impact of salvage therapy over the study period. Therefore, the efficacy of modern salvage treatments represents a plausible primary explanation for the DFS-OS dissociation observed here. This underscores that while VPC status identifies patients with a lower risk of early recurrence, achieving a survival advantage in the era of effective salvage therapy may require strategies that prevent recurrence more durably.

In contrast to some previous studies that demonstrated worse prognosis in patients with VPC+ (26, 29, 30), our study found that VPC- was associated with worse DFS. This discrepancy may be attributed to differences in study design, patient selection, and the specific criteria used to define VPC. Our study excluded patients with VPI, focusing solely on early-stage lung adenocarcinoma, which may have different biological behavior compared to more advanced stages. Additionally, our use of propensity score matching (PSM) to balance confounding factors may have influenced the observed outcomes.

Notably, we did not account for adjuvant therapies in our analysis, which might have restricted the comprehensiveness of our findings, particularly when assessing the relationship between VPC and prognosis. Pleural indentation has been linked to adverse outcomes in NSCLC, as it may heighten the risk of invasion into the lymph-rich visceral pleura, thereby facilitating tumor dissemination (31, 32). Additionally, pleural attachment has been identified as a risk factor for local recurrence after radiotherapy, as well as a risk factor for lower survival rates in lung adenocarcinoma (11, 32–34). However, our study did not differentiate between the various features of VPC; thus, it did not emphasize the significance of pleural indentation. Pre-treatment CT showing pleural attachment has predictive value.

The correlation between radiographic and pathological findings further highlights this critical distinction. Kim et al. (35) reported that CT-defined pleural contact demonstrated low positive predictive value (44%–56%) for pathological VPI and lacked independent prognostic significance for DFS (P > 0.05). Consistent with these observations, Hsu et al. (9) identified that only specific patterns of pleural retraction, notably type 2 pleural retraction characterized by linear traction with soft tissue components at the pleural terminus, could predict VPI with 71% accuracy. In our multicenter cohort, VPC encompassed a spectrum of features, including pleural retraction, pleural tail sign, pleural attachment, and pleural indentation. These radiographic manifestations likely represent localized stromal reactions rather than invasive tumor fronts, explaining the DFS advantage in VPC+ subgroups.

The marginal significance of DFS (P = 0.028) might be due to the limited sample size and the relatively small number of events. Unlike VPI, which is well - studied and recognized as a poor prognostic factor, VPC remains largely unexplored. Our findings indicate that VPC might represent a distinct biological interaction between the tumor and pleura that does not necessarily progress to invasion.

This interpretation aligns with Yang et al.’s (36) detailed radiographic–pathological correlation, which described pleural retraction signs as thick linear tractions with soft tissue components at the pleural margin, often accompanied by tumor-induced pleural buckling, findings strongly associated with VPI on final pathology. However, current controversies persist regarding the CT-based morphological criteria for T-staging. No consensus has been established on whether pleural contact warrants T-stage upstaging (37). This uncertainty stems from the persistent challenges in establishing a definitive radiographic–pathological correlation, as CT evidence of pleural contact or retraction cannot reliably determine the pathological T-stage (10). Notably, while radiographic VPC may indicate the tumor–pleural interaction, our study corroborates the existing literature, suggesting that these findings frequently represent reactive fibroelastotic changes rather than true pleural penetration (9, 35, 36). These observations have critical therapeutic implications.

The influence of nodule characteristics on VPC and VPI is significant. Prior to PSM, statistically significant differences in VPC were observed across different nodule types, including ground-glass, mixed, and solid nodules (P < 0.001). For pGGN, existing studies have demonstrated that pathological VPI does not occur without solid components or pleural changes (9, 23, 38). This indicates that pleural changes in lesions with pGGN are mostly benign interactions.

The situation becomes more complex with mGGN and SN. Previous studies have highlighted pleural retraction as a predictor of VPI in part-solid lung cancers, but the relatively low consolidation ratio in these lesions often results in subtle pleural retraction signs (38, 39). In the present study, 75% of VPC+ nodules were part-solid or solid, which are frequently associated with invasive adenocarcinoma components. However, accompanying pleural fibrosis may modulate the behavior of the tumor. A previous study suggested that stromal fibrosis promotes extracellular matrix remodeling, creating a physical barrier that impedes tumor cell migration (37). This is consistent with the absence of VPI in pGGN with pleural changes (9, 40), as their fibrotic reactions may suppress invasive progression. Conversely, VPI+ tumors can bypass these barriers by breaching the elastic layer and entering subpleural lymphatics, thereby enabling systemic dissemination (41, 42). It should be noted that these mechanisms are based on assumptions about the physical movement of malignant cells rather than being based on validated metastatic processes, which are considered far more complex (23, 43).

Yang et al. (13) demonstrated that VPI significantly impacts both OS and recurrence-free survival in patients with tumors >1 cm in size or a CTR of >50%, whereas VPI shows no prognostic significance in tumors ≤1 cm or with a CTR of ≤50%. These findings highlight the critical clinical relevance of VPI to the proportion of solid components and solid sizes. However, our study used a simplified categorization of pGGN, mGGN, and SN without detailed CTR stratification. The subgroup analysis revealed no significant differences in OS (P = 0.730) or DFS (P = 0.150) among these groups. This suggests that VPC may have distinct prognostic implications compared to VPI, which warrants further investigation in larger cohorts with detailed CTR stratification.

Nodule features exert differential impacts on VPC and VPI. VPC in ground-glass nodules predominantly correlates with benign pathological changes, while VPC in mixed/solid nodules may involve complex interactions requiring further study. These distinctions have prognostic implications for DFS and OS.

This study has several strengths. First, PSM balanced potential confounders, enhancing the comparability of the VPC+ and VPC− groups. Second, the multicenter design increased the external validity by incorporating data from two centers. Third, robust statistical methods, including Cox proportional-hazards regression and Kaplan–Meier analyses, were used to evaluate the influence of VPC on DFS and OS. Finally, our results highlight VPC as a potential prognostic indicator for DFS in early-stage lung adenocarcinoma, offering a valuable direction for future research.

However, this study also has several limitations. The retrospective design may have introduced selection and information biases, potentially affecting the generalizability of the findings. Data collection from medical records could have led to incomplete or inaccurate data, particularly for VPC assessments that rely on imaging and pathological reports, thus introducing subjectivity. The broad definition of VPC, encompassing multiple imaging features, was not stratified by subtype and lacked formal inter-rater reliability assessment, potentially introducing variability in progress and prognostic conclusions. While PSM mitigates some confounding, it cannot eliminate all biases. The small sample size, especially the limited number of VPC+ cases, may have reduced statistical power. Additionally, information on adjuvant therapies was not collected, so their potential impact on DFS and OS could not be evaluated. These limitations underscore the need for larger prospective studies to validate our findings.

In conclusion, our findings suggest that VPC status may relate to DFS in patients with stage IA lung adenocarcinoma, but not to OS. This implies that VPC might be a valuable factor for risk assessment and postoperative follow-up planning in patients with early-stage lung adenocarcinoma. However, owing to the retrospective nature of the study, our results require cautious interpretation. More research, especially prospective studies with larger cohorts, is needed to verify the role of VPC as a prognostic marker and define how VPC assessment could help to manage stage IA lung adenocarcinoma.

## Data Availability

The raw data supporting the conclusions of this article will be made available by the authors, without undue reservation.
